# Survival from adult leukaemia in England and Wales up to 2001

**DOI:** 10.1038/sj.bjc.6604609

**Published:** 2008-09-23

**Authors:** B Rachet, E Mitry, A Shah, N Cooper, M P Coleman

**Affiliations:** 1 Cancer Research UK Cancer Survival Group, Non-Communicable Disease Epidemiology Unit, Department of Epidemiology and Population Health, London School of Hygiene and Tropical Medicine, Keppel Street, London WC1E 7HT, UK; 2 Département d’Hepatogastroentérologie et Oncologie Digestive, Centre Hospitalo-Universitaire Ambroise-Paré, 9 avenue Charles de Gaulle, Boulogne F-92100, France; 3 Social and Health Analysis and Reporting Division, Office for National Statistics (Room FG/114), 1 Myddelton Street, London EC1R 1UW, UK

The leukaemias are a biologically and clinically diverse group of malignancies originating from precursors of the various blood cell series. Advances in basic science and cell surface markers in the last 20 years have led to revised clinical classifications of the leukaemias, as with the lymphomas (Percy *et al*., 1984), but these advances were not all reflected in the first two editions of the International Classification of Diseases for Oncology (ICD-O) (Percy *et al*, 1990; World Health Organisation, 1976), used by cancer registries world-wide to encode the morphology of neoplasms for robust international comparisons. The third edition of ICD-O incorporates these changes, but was introduced too recently to affect these data (Fritz *et al*, 2000). As a result, population-based data series from cancer registries cannot always be used to address clinical questions about trends in outcome for recently defined disease subgroups, as systematic realignment of older data to the more specific recent categories may be impossible. Acute and chronic myeloid leukaemia, chronic lymphoid leukaemia (CLL) and monocytic leukaemia account for 90% of leukaemias in adults in England and Wales. Acute lymphocytic leukaemia is mainly a disease of childhood. Information on survival for all adult leukaemias combined remains of some interest; however, even though the results are a weighted average of socioeconomic patterns and trends in survival for the different types of leukaemia.

Approximately 6200 adults are diagnosed with a leukaemia each year in England and Wales, some 3% of all malignancies. Incidence is marginally higher among more affluent groups, and approximately 30% higher in men overall, but the male excess is more marked for CLL. Incidence has been increasing only slowly since the 1970s, mostly among the elderly (Quinn *et al*., 2001). Geographic variation in incidence has not been marked.

Benzene and ionising radiation are well-established causes of leukaemia, although organic solvents, some viruses (IARC, 1996; Lynge *et al*., 1997; Stellman, 1998) and some antineoplastic drugs are also leukaemogenic (Kaldor *et al*., 1990). Nevertheless, all these potential causes explain only a small proportion of incident cases.

Of nearly 73 000 patients registered with a leukaemia in England and Wales during the period 1986–1999, only approximately 57 000 (78.3%) could be included in survival analyses. As many as 16% of patients were excluded from analysis because their dates of diagnosis and death were identical: this proportion was exceeded only for tumours of the lung and pancreas (Coleman *et al*., 2004). In these data, it was not possible to distinguish patients who died on the day of diagnosis (true zero survival) from those whose date of diagnosis was simply unknown, because they were registered from a death certificate only (DCO), but it is likely that the great majority of such cases were DCOs. The vital status of another 2% of patients was unknown on 5 November 2002, when the data were extracted for analysis, and leukaemia was not the first primary malignancy for a further 3.5% of patients; both groups were also excluded from analysis.

After a steady long-term increase, the incidence of leukaemia in England and Wales reached a plateau in the early 1990s at approximately 10 per 100 000 per year in men and 7.5 in women. The rise in incidence was less steep among more deprived groups, leading to lower incidence than among the more affluent groups of the population, particularly for men.

## Survival trends

**Figure 1:**
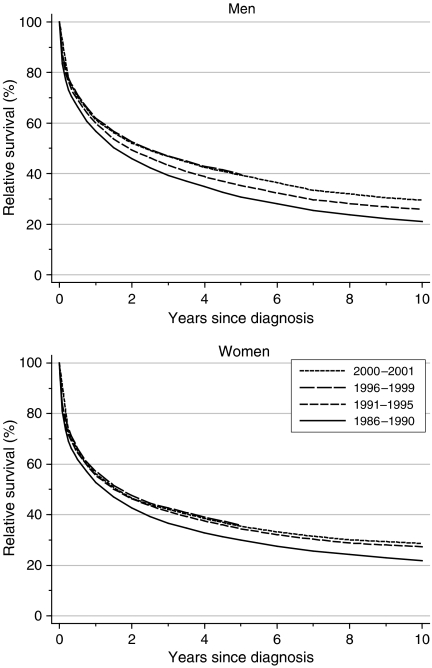
Relative survival (%) up to 10 years after diagnosis by sex and calendar period of diagnosis: England and Wales, adults (15-99 years) diagnosed during 1986–1999 and followed up to 2001. Survival estimated with cohort or complete approach (1986–1990, 1991–1995, 1996–1999) or hybrid approach (2000–2001) (see Rachet *et al*, 2008).

For leukaemia patients diagnosed at the end of the 1990s, relative survival at 1 year was approximately 60%, whereas 5-year survival was 36% (women) or 40% (men; Table 1). Among men, 5-year survival increased significantly by approximately 5% every 5 years between 1986 and 1999, after adjustment for changes in the distribution of incident cases by deprivation category (Figure 1). For women, increases in survival since the late 1980s have been smaller and the pace of increase in survival every 5 years was not statistically significant. Survival among women is generally lower than for men, although 10-year survival is similar in the two sexes.

Hybrid analysis of the survival probabilities observed during 2000–2001 (Brenner and Rachet, 2004) reflects the continuing increase, but even for patients diagnosed around that time, relative survival at 5 and 10 years will still only reach approximately 40 and 30%, respectively.

## Deprivation

**Figure 2:**
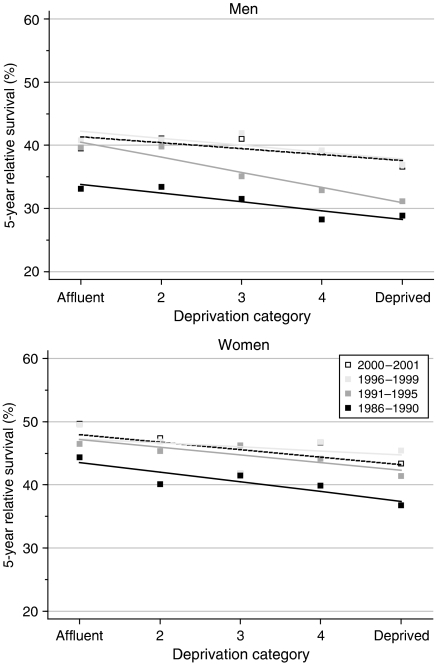
Trends in the deprivation gap in 5-year relative survival (%) by sex and calendar period of diagnosis: England and Wales, adults (15–99 years) diagnosed during 1986–1999 and followed up to 2001.

The deprivation gap in survival from leukaemia in adults is consistently and significantly in favour of the more affluent groups (Table 2, Figure 2). The fitted difference between the most affluent and most deprived groups in survival up to 5 years has been at least 5%, with slightly smaller differences in 10-year survival. For adults diagnosed with leukaemia during the late 1990s, 1-year survival was approximately 6% higher for the most affluent fifth of the adult population than for the most deprived fifth, in both sexes, with 5-year survival 3–4% higher.

For the cohort of men diagnosed with leukaemia during the early 1990s, the deprivation gap in survival at 1, 5 and 10 years after diagnosis seemed substantially wider than for men diagnosed in the late 1980s, but over the whole 14-year period 1986–1999, no consistent change in the deprivation gradient occurred. Among women, there is some evidence of shrinkage of the deprivation gradient in survival, particularly at 5 years (Table 2).

Short-term predictions based on hybrid analysis to predict the likely socioeconomic differences in survival for patients diagnosed during 2000–2001 do not suggest any imminent reduction in the deprivation gap (Table 2).

## Comment

The main limitation of these analyses is that population-based survival trends could not be produced for the specific subtypes of leukaemia defined by recent clinical classifications, although overall survival trends among adults have been broadly similar for all the main types of leukaemia (Berrino *et al*., 1995, 1999, 2003; Coleman *et al*., 1999, 2004).

Despite therapeutic progress, however, the trends and socioeconomic patterns in survival from leukaemia in adults provide a striking contrast with those for leukaemia in children. Survival from all leukaemias combined in adults has improved steadily, but not rapidly, reaching approximately 40% 5-year survival for adults diagnosed by the end of the 1990s, and the deprivation gradient in survival is marked and persistent for both men and women.

For children with leukaemia, in contrast, the increase in survival since the 1970s has been dramatic, 5-year survival reaching 80% by the end of the 1990s. Furthermore, there is no significant socioeconomic gradient in survival, whether for all the leukaemias combined or for acute lymphoblastic leukaemia in particular (Stiller and Eatock, 1994; Coleman *et al*., 1999; Schillinger *et al*., 1999).

The disparities in the trends and socioeconomic inequalities in survival from leukaemia between children and adults are remarkable, and they merit further exploration.

## Figures and Tables

**Table 1:** Trends in relative survival (%) by sex, time since diagnosis and calendar period of diagnosis: England and Wales, adults (15–99 years) diagnosed during 1986–1999 and followed up to 2001

		**Calendar period of diagnosis^a^**									
		**1986–1990**		**1991–1995**		**1996–1999**		**Average change (%) every 5 years^b^**		**Prediction^c^ for patients diagnosed during 2000–2001**	
**Time since diagnosis**		**Survival (%)**	**95% CI**	**Survival (%)**	**95% CI**	**Survival (%)**	**95% CI**	**Survival (%)**	**95% CI**	**Survival (%)**	**95% CI**
1 year	Men	**56.7**	(55.6, 57.7)	**60.0**	(59.1, 60.9)	**61.9**	(60.9, 62.9)	**2.9** ^**^	(1.0, 4.9)	**61.3**	(59.8, 62.7)
	Women	**52.8**	(51.7, 53.9)	**55.6**	(54.5, 56.6)	**57.3**	(56.1, 58.4)	**1.5**	(−0.7, 3.7)	**56.2**	(54.6, 57.9)
5 years	Men	**30.7**	(29.6, 31.7)	**35.3**	(34.3, 36.3)	**39.7**	(38.4, 41.0)	**4.7** ^**^	(2.4, 7.0)	**39.4**	(37.8, 41.0)
	Women	**30.0**	(28.9, 31.1)	**34.5**	(33.3, 35.6)	**35.9**	(34.4, 37.3)	**1.7**	(−0.8, 4.2)	**35.4**	(33.7, 37.1)
10 years	Men	**21.1**	(20.1, 22.1)	**25.8**	(24.6, 27.0)			**8.4** ^**^	(4.4, 12.5)	**29.5**	(27.8, 31.2)
	Women	**21.8**	(20.7, 22.9)	**27.3**	(26.0, 28.6)			**5.2** ^*^	(0.8, 9.7)	**28.6**	(26.8, 30.4)

CI=confidence interval.

a Survival estimated with cohort or complete approach (see Rachet *et al*, 2008).

b Mean absolute change (%) in survival every 5 years, adjusted for deprivation (see Rachet *et al*, 2008).

c Survival estimated with hybrid approach (see Rachet *et al*, 2008).

^*^P<0.05; ^**^P<0.01.

**Table 2:** Trends in the deprivation gap in relative survival (%) by sex, time since diagnosis and calendar period of diagnosis: England and Wales, adults (15–99 years) diagnosed during 1986–1999 and followed up to 2001

		**Calendar period of diagnosis^a^**									
		**1986–1990**		**1991–1995**		**1996–1999**		**Average change (%) every 5 years^b^**		**Prediction^c^ for patients diagnosed during 2000–2001**	
**Time since diagnosis**		**Deprivation gap (%)**	**95% CI**	**Deprivation gap (%)**	**95% CI**	**Deprivation gap (%)**	**95% CI**	**Deprivation gap (%)**	**95% CI**	**Deprivation gap (%)**	**95% CI**
1 year	Men	−5.7**	(−8.7, −2.8)	−9.5**	(−12.2, −6.8)	−6.0**	(−9.0, −3.1)	−**0.2**	(−2.4, 2.0)	−6.0**	(−10.2, −1.9)
	Women	−7.8**	(−11.2, −4.5)	−6.4**	(−9.5, −3.3)	−5.8**	(−9.1, −2.5)	**1.1**	(−1.4, 3.6)	−9.2**	(−13.9, −4.5)
5 years	Men	−5.5**	(−8.6, −2.5)	−9.6**	(−12.5, −6.7)	−4.4*	(−8.3, −0.5)	**0.0**	(−2.5, 2.6)	−**3.8**	(−8.3, 0.8)
	Women	−6.1**	(−9.4, −2.8)	−4.9**	(−8.1, −1.7)	−**2.5**	(−6.7, 1.6)	**1.8**	(−0.9, 4.6)	−**4.8**	(−9.7, 0.2)
10 years	Men	−**2.3**	(−5.3, 0.7)	−7.2**	(−10.7, −3.8)			−4.9*	(−9.5, −0.3)	−**2.4**	(−7.1, 2.4)
	Women	−3.7*	(−6.9, −0.4)	−**3.3**	(−7.1, 0.4)			**0.3**	(−4.7, 5.3)	−**3.3**	(−8.5, 1.9)

CI=confidence interval.

a Survival estimated with cohort or complete approach (see Rachet *et al*, 2008).

b Mean absolute change (%) in the deprivation gap in survival every 5 years, adjusted for the underlying trend in survival (see Rachet *et al*, 2008).

c Survival estimated with hybrid approach (see Rachet *et al*, 2008).

^*^P<0.05; ^**^P<0.01.
